# Effect of cataract surgery on risk of falls or fractures in Medicare beneficiaries with cataracts

**DOI:** 10.1093/geroni/igaf122.1620

**Published:** 2025-12-31

**Authors:** Atalie Thompson, Lindsey Abdelfattah, Emilie Duchesneau, Amol Joshi, Amresh Hanchate

**Affiliations:** Wake Forest University School of Medicine, Winston-Salem, North Carolina, United States; Wake Forest University School of Medicine, Winston-Salem, North Carolina, United States; Wake Forest University School of Medicine, Winston-Salem, North Carolina, United States; Wake Forest University School of Business, Winston-Salem, North Carolina, United States; Wake Forest University School of Medicine, Winston-Salem, North Carolina, United States

## Abstract

Falls and fractures are a significant public health concern linked to visual impairment. Cataracts are the most common cause of visual impairment in older adults, and can be corrected with cataract surgery. We evaluated whether cataract surgery reduces the one-year risk of falls/fractures in Medicare beneficiaries. Using 2019-2021 claims data from a nationally representative cohort of 1 million Medicare fee-for-service enrollees (65+ years), we identified those with an ICD-10 diagnosis of cataract in 2019. Cataract surgery in 2020 was identified using procedure codes and participants were followed for one year from the surgery date; individuals with cataracts who did not receive surgery were included as controls and assigned a random pseudo-index date. We used propensity score weighting to standardize covariates (demographics, frailty, comorbidities, eye diseases, socioeconomic indicators) among controls (N = 240,164) to resemble those with cataract surgery (N = 16,957). We applied a propensity score weighted linear regression model to estimate the impact of surgery on 1-year risk of falls/fractures. Without surgery, the estimated likelihood of falls/fractures was 8.92% ((95% CI:8.64-9.21%), p < 0.001). The estimated effect of surgery was a reduction by -0.32 percentage points ((95% CI: 1.22-0.59), p = 0.494). Sensitivity analyses treating death as a competing risk, adjusting for prior falls/fractures, and evaluating number of falls/fractures yielded similar results. Thus, cataract surgery did not significantly impact the one-year risk of having falls/fractures in Medicare beneficiaries. Future studies should evaluate fall risk over a longer follow-up period or the impact of surgery on self-reported falls not captured by the healthcare system.

